# Biophysical Basis of Alpha Rhythm Disruption in Alzheimer’s Disease

**DOI:** 10.1523/ENEURO.0293-19.2020

**Published:** 2020-04-28

**Authors:** Rohan Sharma, Suhita Nadkarni

**Affiliations:** Division of Biology, Indian Institute of Science Education and Research, Pune 411008, India

**Keywords:** alpha rhythm, Alzheimer’s disease, HCN, thalamic network

## Abstract

Occipital alpha is a prominent rhythm (∼10 Hz) detected in electroencephalography (EEG) during wakeful relaxation with closed eyes. The rhythm is generated by a subclass of thalamic pacemaker cells that burst at the alpha frequency, orchestrated by the interplay of hyperpolarization-activated cyclic nucleotide-gated channels (HCN) and calcium channels in response to elevated levels of ambient acetylcholine (ACh). These oscillations are known to have a lower peak frequency and coherence in the early stages of Alzheimer’s disease (AD). Interestingly, calcium signaling, HCN channel expression and ACh signaling, crucial for orchestrating the alpha rhythm, are also known to be aberrational in AD. In a biophysically detailed network model of the thalamic circuit, we investigate the changes in molecular signaling and the causal relationships between them that lead to a disrupted thalamic alpha in AD. Our simulations show that lowered HCN expression leads to a slower thalamic alpha, which can be rescued by increasing ACh levels, a common therapeutic target of AD drugs. However, this rescue is possible only over a limited range of reduced HCN expression. The model predicts that lowered HCN expression can modify the network activity in the thalamic circuit leading to increased GABA release in the thalamus and disrupt the calcium homeostasis. The changes in calcium signaling make the network more susceptible to noise, causing a loss in rhythmic activity. Based on our results, we propose that reduced frequency and coherence of the occipital alpha rhythm seen in AD may result from downregulated HCN expression, rather than modified cholinergic signaling.

## Significance Statement

Aggregation of amyloid-β, modified expression of ion channels and alterations in calcium signaling are hallmarks of Alzheimer’s disease (AD) neurons. Separately, changes in the alpha rhythm is often an early observation in AD patients. We use a realistic computational model of the thalamus to elucidate the causal links between molecular changes in AD and their effect on alpha rhythm. Our model demonstrates that pathology of hyperpolarization-activated cyclic nucleotide-gated channels (HCN), crucial for alpha generation, alters calcium signaling, modifies excitation-inhibition balance in the thalamus makes the network more sensitive to noise. Our model, when seen in conjunction with diverse experimental data, posits a causal relationship between the formation of amyloid-β plaques, the downregulation of HCN channels and aberrations in the occipital alpha rhythm.

## Introduction

Brain rhythms play a vital role in coordinating and organizing neuronal computations across various anatomic regions of the brain. These rhythms range from a fraction of a hertz (delta) to over a hundred hertz (high gamma) and have been implicated in attention, spatial navigation, and memory consolidation (Buzsaki, 2006).

The alpha rhythm (7.5–12.5 Hz) in particular is associated with attention and semantic orientation (Rihs et al., 2007) and is seen to be modulated by changes in behavior (Buzsaki, 2006). In attention and discrimination tasks, the coherence in the alpha band measured from somatosensory and motor cortices decreases along with a concomitant increase in the power surrounding the 20-Hz band (Haegens et al., 2011). This indicates that coherence and frequency changes are functionally relevant attributes of the alpha rhythm. Apart from somatosensory and motor cortices, alpha band oscillations have also been observed in other brain regions like the prefrontal cortex (Halgren et al., 2002), auditory cortex (Tiihonen et al., 1991), and over the occipital lobe (Hughes and Crunelli, 2005). Alpha Rhythm activity over the occipital lobe, observed during relaxed, closed eye wakefulness (Berger, 1929; Hughes and Crunelli, 2005), is the focus of the present study.

Positron emission tomography (PET) and electroencephalography (EEG) recordings in humans indicate that the occipital alpha emerges from the interplay between cortical and thalamic neuronal networks (Schreckenberger et al., 2004). Upon muscarinic cholinergic activation, the *in vitro* local field potential (LFP) from the thalamus exhibits alpha band activity (Lőrincz et al., 2008). Additionally, application of muscarinic cholinergic antagonists to the thalamus [specifically to the lateral geniculate nucleus (LGN)] reduce alpha band frequency and power over the occipital region, as seen in EEG recordings of cats (Lőrincz et al., 2009). These alpha band oscillations over the occipital region, observed in EEG, are highly synchronized with the alpha band oscillations in the thalamic LFP (Hughes et al., 2011). Together, these studies indicate (1) a causal link between thalamic alpha oscillations and the alpha rhythm activity seen in EEG recordings over the occipital lobe and (2) cholinergic modulation as a trigger of thalamic alpha. While the alpha rhythm is a complex and ubiquitous phenomenon, observed over different anatomic areas during diverse behavioral states and arising from complex interactions between thalamocortical circuits, we restrict our investigation to the alpha rhythm originating in the thalamus during closed-eye relaxation and measured in EEG over the occipital region.

Patients of Parkinson’s disease, Alzheimer’s disease (AD), and other forms of dementia show distinct changes in their EEG recordings (Friston et al., 2015). In particular, lowering of the average dominant occipital alpha (DOA) frequency in EEG recordings is seen across a population of AD patients (Vitiello, 1989; Crunelli et al., 2015; Blinowska et al., 2017). As mentioned before, the frequency and power of both the thalamic alpha rhythm (observed in the LFP) and the occipital alpha rhythm (recorded in EEG) are modulated by the concentration of ambient acetylcholine (ACh; Lőrincz et al., 2008, 2009; Hughes et al., 2011). A class of drugs that inhibit the breakdown of ACh (acetylcholinesterase inhibitors), and therefore augment its resting levels, can provide temporary symptomatic relief in AD. They are also shown to increase occipital alpha rhythm frequency and coherence (Babiloni et al., 2013). These observations, along with recent studies which show that thalamic degeneration precedes symptoms of cognitive decline in AD (De Jong et al., 2008; Aggleton et al., 2016), suggest a link between AD, the thalamic alpha rhythm and cholinergic signaling.

We investigate different biochemical changes associated with AD using a realistic computational model of the thalamic network (Vijayan and Kopell, 2012) that generates the alpha rhythm. The network consists of thalamocortical cells (TCs), reticular cells (REs) and specialized TC cells, the so-called HTC cells due to the expression of certain high-threshold calcium channels (more details in methods). Individual HTC cells generate intrinsic oscillations due to the interplay between the high threshold calcium current and hyperpolarization-activated cyclic nucleotide-gated channels (HCN) channels. Consistent with the extant literature (Hughes et al., 2011), thalamic alpha in our model arises from this intrinsic activity of HTC neurons which synchronize via gap-junctions. These cells fire at ∼10 Hz when the ambient level of ACh is high enough to activate muscarinic ACh receptors (mAChRs; Lőrincz et al., 2008). Amyloid-β plaques are a characterizing feature of AD. Separately, lowered HCN channel expression in brain slices of AD patients was shown to cause an increase in the production of amyloid-β peptide (Saito et al., 2012). Apart from changes in cholinergic signaling, these observations provide an additional correspondence between the alpha rhythm and AD, mediated by HCN channels and calcium signaling. Our study examines the causal links between the biochemical changes observed in AD and quantifies the changes in the thalamic network dynamics that underlie a modified thalamic alpha rhythm.

## Materials and Methods

### The thalamic network

**Figure 1.**
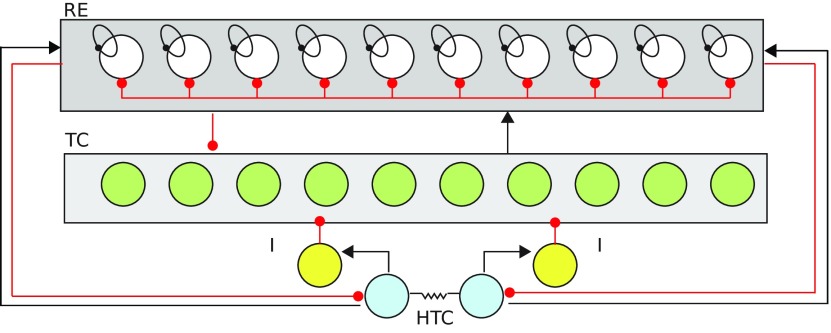
Thalamic network involved in alpha generation. The network described here consists of the reticular cells (REs), the thalamocortical neurons (TC cells) and high-threshold TCs (HTC). The RE cells (white) mutually inhibit each other and inhibit all of the TC cells (green). TC cells send excitatory connections to all of the RE cells. There are no direct mutual connections between TC cells. The HTC cells (blue), responsible for generating the α rhythm, inhibit the TC cells via sending an excitatory drive to the inhibitory-interneurons (yellow). The ratio of the RE to TC cells is 1:1 and 20% of the TC cells are HTC cells. The inhibitory interneurons are modeled implicitly as an inhibitory synapse from HTCs to TCs with a time delay. Gap-junctions between the HTC cells enable synchronized bursting.

The alpha rhythm originates in HTC cells that innervate and receive input from the rest of the thalamic network. The HTC neurons are synchronized to each other via gap-junctional connectivity. Our model is a scaled down motif of the thalamic network (Fig. 1) and based on the model proposed by Vijayan and Kopell (2012). The model is the canonical model (Destexhe and Sejnowski, 1997) of the thalamocortical circuitry, with the addition of a high-threshold T-type calcium current in 20% of the TC cells (HTC; Fig. 1). It consists of two HTC cells coupled via gap-junctions that provide an excitatory drive to RE cells and an inhibitory drive to TC cells. The TC cells are excitatory and connect to all RE cells but not to each other or HTC cells. The RE cells are GABAergic and inhibit every other cell in the network including other RE cells (Fig. 1). The HTC cells receive a white-noise input with zero mean which was implemented using the Euler–Maruyama method. The TC and RE neurons receive a Poisson-distributed train of excitatory and inhibitory impulses. The activation of the mAChRs is modeled by lowering the potassium leak conductance (McCormick and Prince, 1986; Vijayan and Kopell, 2012).

### Code accessibility

Simulations were performed on a high-performance computing cluster (HP PROLIANT SL230s Gen8 as compute nodes, each with two CPUs containing 10 cores each; CPU: Intel(R) Xeon(R) CPU EV-2860 v2 2.80 GHz) with 1464 processing units housed in IISER Pune. We used an in-house Computational Neuroscience library written in C++ to perform the simulations. The code described in this paper is freely available at https://github.com/rhnshrma/ENEURO0293-19.2020. The time step of each simulation was taken to be 0.01 ms.

### Quantitative details of HTC cells

Our investigation primarily concerns HTC cells as they are the locus of all manipulations. The details of TC cells, RE cells and synapses are presented later.

(1) $$C\frac{dV_{HTC}}{dt}=-I_{Na}-I_{K}-I_{TLT}-I_{THT}-I_{L}-I_{H}-I_{AHP}-I_{GJ}-I_{GABA_{A}}-I_{GABA_{B}}+I_{APP}.$$

Potassium current:

$$I_{K}=g_{k}n^{4}(V-E_{K})\;\;\;\;\;\;\frac{dn}{dt}=\frac{n_{\infty }(V)-n}{\tau_{n}(V)},$$

here:

$$n_{\infty }(V)=\frac{\alpha_{n}(V)}{\alpha_{n}(V)+\beta_{n}(V)}\;\;\;\;\tau_{n}(V)=\frac{1}{\alpha_{n}(V)+\beta_{n}(V)},$$

where:

$$\alpha_{n}=\frac{0.032(15-V_{t})}{exp((15-V_{t})/5.0)-1}\beta_{n}=0.5exp((10-V_{t})/40).$$

$$V_{t}=V_{HTC}+25,\;g_{K}=10mS/cm^{2}\;and\;E_{K}=-100mV.$$

Sodium current:

The $m_{\infty };\tau_{m};h_{\infty };and;\tau_{h}$ have equations identical to the *n_∞_* and the *τ_n_* of the potassium gate n.

$$I_{Na}=g_{Na}m^{3}h(V-E_{Na}),\;\frac{dm}{dt}=\frac{m_{\infty }(V)-m}{\tau_{m}(V)},\frac{dh}{dt}=\frac{h_{\infty }(V)-h}{\tau_{h}(V)},$$

where:

$$\begin{array}{l} {\mathrm{alpha}}_{m}=\frac{0.32(13-V_{t})}{exp((13-V_{t})/4.0)-1}\beta_{m}=\frac{0.28(V_{t}-40)}{exp((V_{t}-40)/5)-1}\\ {\mathrm{alpha}}_{h}=0.128exp((17-V_{t})/18)\beta_{h}=\frac{4}{exp((40-V_{t})/5)\;+\;1} \end{array}$$

$$V_{t}=V_{\mathrm{HTC}}+25,\;g_{\mathrm{Na}}=90mS/cm^{2}\;\mathrm{and}E_{\mathrm{Na}}=+50\;mV.$$

Low-threshold calcium current:

$$I_{TLT}=g_{TLT}m^{2}h(V-E_{Ca}),\;m=m_{\infty }(V),\frac{dh}{dt}=\frac{h_{\infty }(V)-h}{\tau_{h}(V)},$$

where:

$$\begin{array}{l} m_{\infty }(V)=\frac{1}{exp(-(57+V_{t})/6.2)+1}\\ h_{\infty }=\frac{1}{1+exp((V_{t}+81.0)/4.0)},\;\tau_{h}=\frac{30.8+\left(\frac{211.4+exp(V_{t}+113.2)/5.0}{1+exp(V_{t}+84.0)/3.2}\right)}{3.74}.\\ \frac{d[Ca]}{dt}=\frac{-10I_{TLT}}{2\times 96489}\;+\;\frac{0.00024-[Ca]}{5.0} \end{array}$$

If the first term is negative, it is set to zero. $g_{TLT}=2mS/cm^{2},\;V_{t}=V_{HTC}+2$ and the reversal potential for calcium is calculated using the Nernst equation.

High-threshold calcium current:

$$I_{THT}=g_{THT}m^{2}h(V-E_{Ca}),\;m=m_{\infty }(V),\;\frac{dh}{dt}=\frac{h_{\infty }(V)-h}{\tau_{h}(V)},$$

,where:

$$\begin{array}{l} m_{\infty }(V)=\frac{1}{exp(-(40.1+V)/3.5)+1}\\ h_{\infty }=\frac{1}{1+exp((V_{t}+62.2)/5.5)},\;\tau_{h}=0.1483exp(-0.09398*V)+5.284exp(0.008855*V).\\ \frac{d[Ca]}{dt}=\frac{-10(I_{TLT}+I_{THT})}{2\times 96489}\;+\;\frac{0.00024-[Ca]}{3.0} \end{array}$$

The first term must be positive, otherwise it is set to zero.

In the model described by Vijayan and Kopell (2012), the calcium concentrations in HTC neurons experienced by the two calcium channels, I*_THT_* and I*_TLT_*, are different. We do not make this additional assumption. In our model, both I_THT_ and I_TLT_ channels, sense the same intracellular calcium. We adjust for this change by using values of g_THT_ and g_Kleak_ which produce stable 10-Hz oscillations in HTC cells.

$g_{THT}=12mS/cm^{2}$ (modified from Vijayan and Kopell, 2012)

*E_Ca_* is calculated using the Nernst equation.

Leak current:

$$I_{L}=g_{L}(V-E_{L})+g_{KL}(V-E_{KL}),$$

where:

$$g_{L}=0.01mS/cm^{2},E_{L}=-70mV,g_{KL}=0.01mS/cm^{2},E_{KL}=-100mV.$$

The effect of ACh is modeled as a lowered potassium leak conductance *g_Kleak_* (the details will be discussed in Results; see Eq. 5; Vijayan and Kopell, 2012; Krishnan et al., 2016).

H-current:

$$I_{H}=g_{h}r(V-E_{h}),\;\frac{dr}{dt}=\frac{r(V)-r_{\infty }}{\tau_{r}(V)},$$

where:

$$r_{\infty }=\frac{1}{1+exp((V+60)/5.5)},\;\tau_{r}=20+\frac{1000}{exp((V+56.5)/14.2)+exp(-(V+74)/11.6)}$$

$$g_{h}=0.36mS/cm^{2},\;and\;E_{h}=-40mV.$$

Calcium-activated potassium current:

$$I_{AHP}=g_{AHP}m^{2}(V-E_{K}),\;\frac{dm}{dt}=\frac{m_{\infty }-m}{\tau_{m}},$$

where:

$$\begin{array}{l} m_{\infty }=\frac{48{\left[Ca\right]}^{2}}{48{\left[Ca\right]}^{2}+0.09}\\ \tau_{m}=\frac{1}{48{\left[Ca\right]}^{2}+0.09} \end{array}$$

$$g_{\mathrm{AHP}}=15mS/cm^{2},\;and\;E_{k}=-100mV.$$

Gap-junction current:

$$I_{GJ}=g_{GJ}(V_{HTC}-V_{post}),$$

where V*_post_* is the membrane potential of the neuron that is connected to this HTC neuron by a gap-junction

$$g_{GJ}=0.003-0.005\;mS/cm^{2}.$$

**Figure 2.**
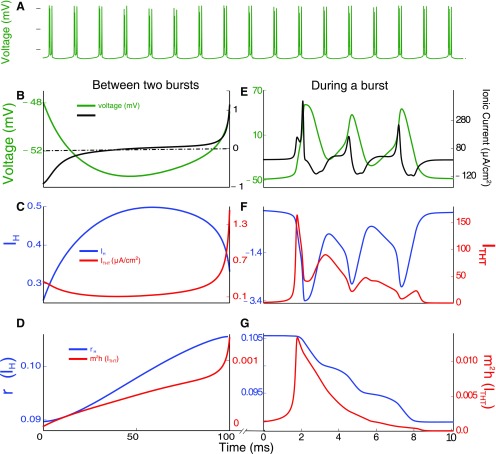
Chronology of events underlying the thalamic alpha. ***A***, The interburst interval determines the 10-Hz rhythm underlying the thalamic alpha. Membrane voltage (green) of a single HTC neuron as it goes through bursts of activity. ***C***, ***D***, Activity of relevant ion channels between the bursts for the duration of a 100-ms time window. ***F***, ***G***, Activity of the same channels during the 10-ms burst of the cell. ***B***, Slowly increasing membrane voltage (green) due to the increase in I_H_ conductance (blue, ***B***) during repolarization of the membrane potential by the potassium current. The total current (black) increases with I_H_. The bursting activity is initiated by activation of I_THT_ current (red, ***E***, ***F***) and terminated by repolarization due to the potassium current. ***C***, The hyperpolarization-activated current I_H_ (blue) increases slowly during membrane repolarization leading to eventual activation of the I_THT_ (red). ***D***, Gating variables of the I_H_ (r_H_, blue) and I_THT_ (m^2^h, red) currents. The high-threshold calcium current gets activated around the ∼90-ms mark. ***E***, The increase in I_THT_ activates the fast sodium channels and triggers a series of APs. Membrane potential (green) and total ionic current in the immediate 10 ms after the activation of I_THT_. This burst of APs is initiated by I_H_ and I_THT_ in that order (left panel). ***F***, The high-threshold calcium current (red) provides a depolarizing impetus that sustains a burst of APs. The I_H_ (blue) current becomes hyperpolarizing above −40-mV membrane voltage but does not contribute much in this phase due to low conductance values compared with the high-threshold calcium. ***G***, The slow decay of the gating variables of the I_H_ (blue) as the membrane potential rises above the activation voltage. The gating variables of I_THT_ (red) slowly deactivate at high voltages during APs.

In reality, the HTC cells receive inputs from various other neurons. Under the diffusion limit of synaptic noise, where the weights are very small and there are a lot of Poissonian synaptic inputs, the synaptic noise can be modeled as Gaussian white noise (Lánská et al., 1994). The HTC cells are also subject to thermal noise, channel conductance fluctuations and channel shot noise (Gerstein and Mandelbrot, 1964). These fluctuations occur at timescales that are much faster than any timescales in the biophysical model. These sources of noise can be modeled cumulatively as a low amplitude Gaussian white-noise. The HTC cells receive a Gaussian distributed white noise through the stochastic Euler-Maruyama integrator (this noise is not present in results shown in Fig. 2):

(2) $$\frac{dV_{HTC}}{dt}=-\Sigma I_{channels}+\sqrt[{}]{dt}\times \xi (t)$$

where *ξ*(*t*) is drawn from a Gaussian distribution with mean 0 and variance 0.1.

### Entropy measure

V_i_ is the membrane voltage of the HTC neurons, where *i* ∈ {1,2}

$$LFP(t)=\frac{\left(V_{1}(t)+V_{2}(t)\right)}{2}.$$

Before performing the discrete fast Fourier transformation (DFFT), the LFP trace is smoothed by taking a simple moving average over a window of 10 ms (which corresponds to 25 discrete points as our observation frequency in 2.5 kHz; Eq. 3), and the mean is subtracted from each data point to make the new mean zero. We smoothed the LFP trace to suppress contributions to the power spectrum from high-frequency noise. The moving average with 10-ms time window will damp contributions to the power spectrum from frequencies above 60 Hz. This is sufficient for our purposes as we are interested in frequencies around the 4- to 30-Hz range. The filtering makes the entropy of the Fourier transform a cleaner measure of aperiodicity.

(3) $$\begin{array}{l} LFP\prime (t_{\text{i}})=\frac{\sum_{j=i-24}^{i}LFP(t_{j})}{25}-\frac{\sum_{j=1}^{N}LFP(t_{j})}{N}\\ F(\omega )=DFFT(LFP\prime (t))\\ p(\omega )=\frac{|F(\omega ){|}^{2}}{\int_{0}^{\infty }|F(\omega ){|}^{2}d\omega } \end{array}$$

*p*(*ω*) is the probability distribution function which is used to calculate the Shanon entropy:

(4) $$entropy=-\sum_{\omega_{i}}p(\omega_{i})log(p(\omega_{i})).$$

#### Quantitative details of TC cells and RE cells

Thalamo-reticular (RE) neurons

(6) $$C\frac{dV_{RE}}{dt}=-I_{Na}-I_{K}-I_{TRE}-I_{L}-I_{GABA_{A}}-I_{AMPA}+I_{EPSP}+I_{IPSP}.$$

Potassium current:

$$I_{K}=g_{k}n^{4}(V-E_{K})\;\;\;\frac{dn}{dt}=\frac{n_{\infty }(V)-n}{\tau_{n}(V)},$$

,here:

$$n_{\infty }(V)=\frac{{\mathrm{alpha}}_{n}(V)}{{\mathrm{alpha}}_{n}(V)+\beta_{n}(V)}\;\;\tau_{n}(V)=\frac{1}{{\mathrm{alpha}}_{n}(V)+\beta_{n}(V)},$$

,where:

$$\alpha_{n}=\frac{0.032(15-V_{t})}{exp((15-V_{t})/5.0)-1}\;\;\beta_{n}=0.5exp((10-V_{t})/40)$$

$$V_{t}=V_{RE}+55,\;g_{K}=10mS/cm^{2}\;andE_{K}=-100mV.$$

Sodium current:

The $m_{\infty };\tau_{m};h_{\infty };and;\tau_{h}$ have equations identical to the *n_∞_* and the *τ_n_* of the potassium gate n.

$$I_{Na}=g_{Na}m^{3}h(V-E_{Na}),\;\frac{dm}{dt}=\frac{m_{\infty }(V)-m}{\tau_{m}(V)},\;\frac{dh}{dt}=\frac{h_{\infty }(V)-h}{\tau_{h}(V)},$$

,where:

$$\begin{array}{l} \alpha_{m}=\frac{0.32(13-V_{t})}{exp((13-V_{t})/4.0)-1}\;\;\beta_{m}=\frac{0.28(V_{t}-40)}{exp((V_{t}-40)/5)-1}\\ {\mathrm{alpha}}_{h}=0.128exp((17-V_{t})/18)\;\;\beta_{h}=\frac{4}{exp((40-V_{t})/5)+1} \end{array}$$

$$V_{t}=V_{\mathrm{RE}}+55,\;\;g_{\mathrm{Na}}=100mS/cm^{2}\;andE_{\mathrm{Na}}=+50\;mV.$$

Calcium current:

$$I_{TRE}=g_{TRE}m^{2}h(V-E_{Ca}),\;\frac{dm}{dt}=\frac{m_{\infty }(V)-m}{\tau_{m}(V)},\;\frac{dh}{dt}=\frac{h_{\infty }(V)-h}{\tau_{h}(V)},$$

,where:

$$\begin{array}{l} m_{\infty }(V)=\frac{1}{exp(-(52+V)/7.4)+1},\;\tau_{m}=0.99+\frac{0.333}{exp((V+27)/10)+exp(-(V+102)/15)}\\ h_{\infty }=\frac{1}{1+exp((V+80.0)/5.0)},\;\tau_{h}=28.307+\frac{0.33}{exp((V+48)/4)+exp(-(V+407)/50)}.\\ \frac{d[Ca]}{dt}=\frac{-10I_{TRE}}{2\times 96489}\;+\;\frac{0.00024-[Ca]}{3.0} \end{array}$$

The first term must be positive, otherwise it is set to zero. $g_{TRE}=2.3mS/cm^{2}$ and the reversal potential for calcium is calculated using the Nernst equation.

Leak current:

$$I_{L}=g_{L}(V-E_{L})+g_{KL}(V-E_{KL}),$$

,where:

$$g_{L}=0.01mS/cm^{2},\;and\;E_{L}=-73mVg_{\mathrm{KL}}=0.08mS/cm^{2},\;and\;E_{\mathrm{KL}}=-100mV.$$

Poisson noise (I*_IPSP_* and I*_EPSP_*):

The applied current is a train of Poisson-distributed excitatory and inhibitory impulses. The details of the same will be discussed in the following section.

### Thalamo-cortical (TC) neurons

(7) $$C\frac{dV_{TC}}{dt}=-I_{Na}-I_{K}-I_{TLT}-I_{L}-I_{H}-I_{GABA_{A}}-I_{GABA_{B}}+I_{EPSP}.$$

Potassium current:

The potassium current follows the same dynamics as the potassium current in HTC cells.

Sodium current:

The sodium current follows the same dynamics as the sodium current in HTC cells.

Low-threshold calcium current:

The low threshold calcium current follows the same dynamics as the low threshold calcium current in HTC cells.

Leak current:

$$I_{L}=g_{L}(V-E_{L})+g_{KL}(V-E_{KL}),$$

,where:

$$g_{L}=0.01mS/cm^{2},E_{L}=-70mV,g_{KL}=0.0028mS/cm^{2},E_{KL}=-100mV.$$

H-current:

$$\begin{array}{l} I_{H}=g_{h}(o+a\times (1-c-o))(V-E_{h}),\;\frac{do}{dt}=0.0001(1.0-c-o)-0.001((1.0-p)/0.01),\\ \frac{dp}{dt}=0.0004(1.0-p)-0.004{\left(\frac{\left[Ca\right]}{0.0002}\right)}^{2},\;\frac{dc}{dt}=\beta_{c}o-\alpha_{c}c \end{array}$$

where:

$$\begin{array}{l} {\mathrm{alpha}}_{c}=\frac{h_{\infty }}{\tau_{s}},\;\beta_{c}=\frac{1-h_{\infty }}{\tau_{s}},\;h_{\infty }=\frac{1}{1+exp((V+75)/5.5)},\\ \tau_{s}=20+\frac{1000}{exp((V+71.5)/14.2)+exp(-(V+89)/11.6)} \end{array}$$

$$g_{L}=0.1mS/cm^{2},\;a=\mathrm{2 a}nd\;E_{h}=-43mV.$$

Poisson noise (I*_EPSP_*):

The applied current is a train of Poisson-distributed excitatory and inhibitory impulses. The details are given below.

Firing rate:

The firing rate shown in Figure 7 (presented later) was obtained by counting the number of times the voltage of the cell crossed 0 mV during the simulation and then dividing that number by the duration of the simulation (∼14 s). The firing rates for all the TC and RE neurons were then averaged for all the cells and all the 5 trials.

Noise:

$$\begin{array}{l} I_{EPSP}=-g_{s}exp(T(t)-t)(V-0)\\ I_{IPSP}=-g_{s}exp(T(t)-t)(V+85) \end{array}.$$

,where:

$$T(t)=min\left\{T_{1},T_{2}.......,T_{n-1},T_{n},.......,|t<T(t)\right\}.$$

The difference between the impulse times, $T_{1},T_{2}....T_{n}$ is an exponentially distributed random variable with a mean of 10 ms for TC and RE cells.

For RE cells:

$g_{s}=0.02mS/cm^{2}$ for EPSPs and $g_{s}=0.015mS/cm^{2}$ for IPSPs.

TC neurons are stimulated with EPSPs with but no IPSPs:

$$g_{s}=1.0mS/cm^{2}.$$

### Quantitative details for synapses

AMPA:

$$I_{AMPA}=g_{AMPA}[R](V-E_{AMPA})\;\frac{d[R]}{dt}=0.98[T](1-[R])-0.180[R].$$

[T] is the transmitter concentration. In response an action potential (AP) the transmitter concentration is increased to 0.5 mM and stays there for 0.3 ms for HTC and 0.5 ms for TC cells. [R] represents the fraction of the receptors that are open.

$$\begin{array}{l} E_{AMPA}=0mV\\ g_{AMPA}:HTC\Rightarrow RE=0.001,\;TC\Rightarrow RE=0.05 \end{array}.$$

GABA_A_:

$$I_{GABA_{A}}=g_{GABA_{A}}[R](V-E_{GABA_{A}})\;\frac{d[R]}{dt}=20[T](1-[R])-0.180[R].$$

[T] is the neurotransmitter concentration. In response an AP thee transmitter concentration is increased to 0.5 mm and stays there for 1.0 ms for HTC and 0.3 ms for RE cells. [R] represents the fraction of the receptors that are open.

$$\begin{array}{l} E_{GABA_{A}}=-85mV,\;\\ g_{GABA_{A}}:HTC\Rightarrow TC=0.4(with\;a\;delay\;of10ms)\\ RE\Rightarrow HTC=0.0002,\;RE\Rightarrow TC=0.002,RE\Rightarrow RE=0.02 \end{array}$$

GABA_B_:

$$\begin{array}{l} I_{GABA_{B}}=g_{GABA_{B}}\left(\frac{{\left[G\right]}^{4}}{{\left[G\right]}^{4}+100}\right)(V-E_{GABA_{B}})\\ \frac{d[G]}{dt}=0.18[R]-0.034[G]\;\frac{d[R]}{dt}=0.09[T](1-[R])-0.0012[R] \end{array}$$

[T] is the neurotransmitter concentration. In response an AP the transmitter concentration is increased to 0.5 mM and stays there for 0.3 ms. [R] represents the fraction of the receptors that are open. [G] is the concentration of the G protein that gets activated on agonization of the receptors.

$$E_{{\mathrm{GABA}}_{B}}=-\mathrm{95}\;mVg_{{\mathrm{GABA}}_{B}}:RE\Rightarrow HTC=0.004{\text{mS}/\text{cm}}^{2},\;RE\Rightarrow TC=0.004{\text{mS}/\text{cm}}^{2}.$$

## Results

### Ionic current dynamics that determine the thalamic alpha timescale

**Figure 3.**
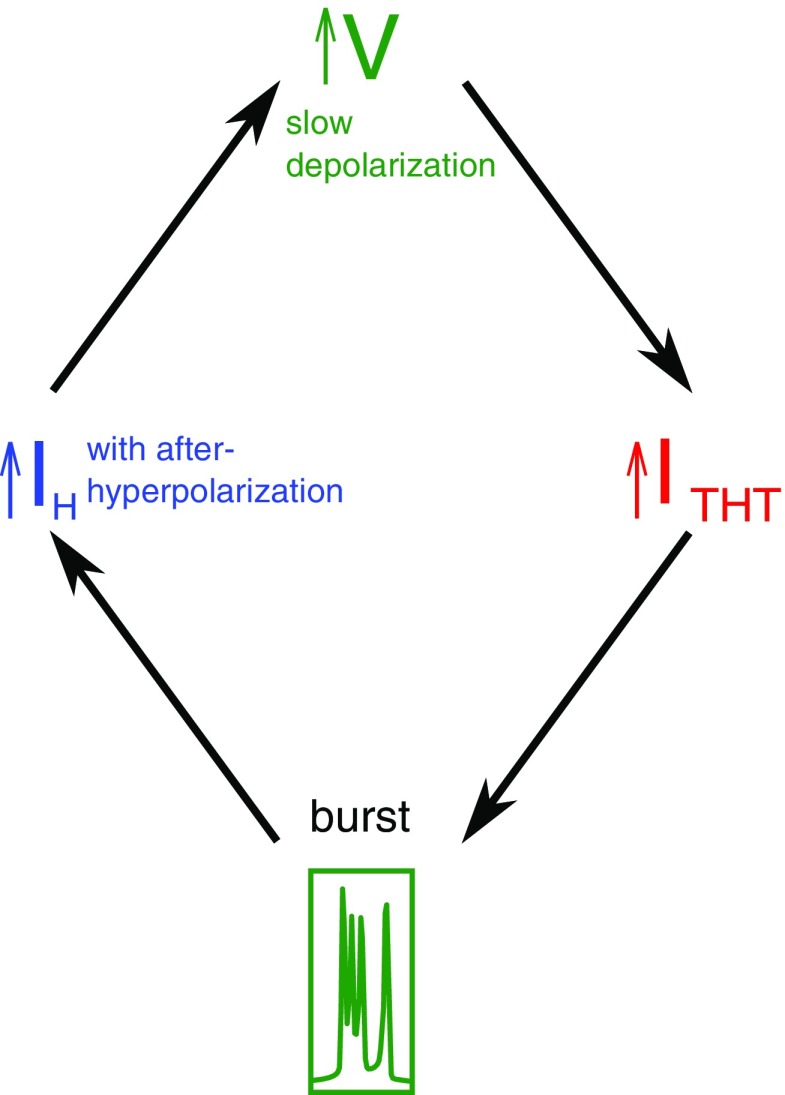
Schematic illustrating the chronology of events. After a burst, the cell membrane hyperpolarizes to subthreshold voltages. This activates the I_H_ current which slowly depolarizes the membrane voltage. This leads to the activation of the I_THT_ current, which in turn, triggers another burst.

We first describe the activity of a single HTC cell (no noise) to elucidate the mechanisms that generate the characteristic 10-Hz timescale of the alpha rhythm. The chronology of events that result in the generation of the rhythm in the presence of ACh is described in Figure 2. High-threshold calcium ion channels and a non-specific (for ions) HCN channel are the ionic components of HTC cells that govern the rhythm. The precision and robustness of this rhythm are determined by the intrinsic dynamics of the gating variables of each current and the interplay between the currents mediated by membrane voltage and calcium. After each burst of APs, the cell undergoes a brief hyperpolarization that activates the HCN channels (I_H_; Fig. 2*B*, blue). This leads to a slow positive inward current (Fig. 2*B*) that steadily depolarizes the cell until it reaches the threshold voltage where calcium channels, I_THT_, open (Fig. 2*B*, red). These calcium channels are activated near instantaneously above the threshold but inactivated over a much slower timescale (Fig. 2*E*,*F*, red). Further, these channels open only within a narrow voltage range (approximately −50 to −10 mV). The calcium current causes a rapid depolarization (Fig. 2*B*), triggering the fast sodium channel leading to a burst of APs (Fig. 2*E*). The slow inactivation gate of the calcium channel leads to the slow decay of the calcium current that eventually terminates the burst of APs (Fig. 2*E–G*). This allows the potassium channel to repolarize the membrane. As the membrane repolarizes, the HCN channels get activated, setting the system up for another cycle (for a summary of these events, please see Fig. 3). The timescale of depolarization determines the interval between bursts and thus the frequency of the alpha rhythm. The depolarization timescale is determined by the magnitude of the I_H_ current, which, in turn, depends on the conductance and expression of HCN channels.

### Effect of varying HCN expression in HTC cells on the thalamic alpha

**Figure 4.**
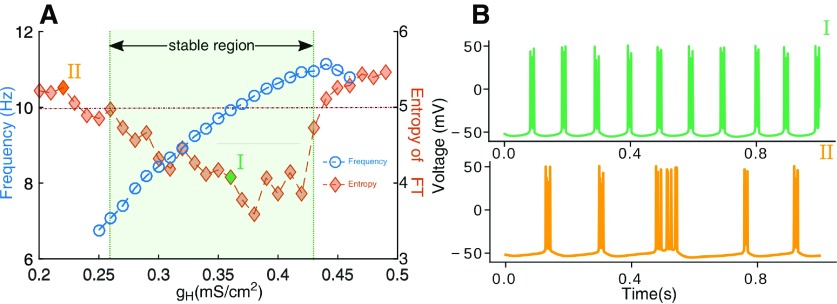
Monotonic dependence of the peak frequency by varying HCN expression. ***A***, The intrinsic oscillation frequency (blue, open circles) of HTC cells shows a monotonic dependence over a limited range of HCN expression (g_H_; marked as the stable region). However, beyond this range continued HCN overexpression (right of the stable region) leads to HTC cells losing periodicity (due to overexcitability). In the other direction (left of the stable region), low levels of HCN expression makes the system more sensitive to noise, resulting in a loss of periodicity again. There exists an optimal regime of g_H_ where bursting is regular. This is depicted as lower entropy (orange, filled diamonds) for a range of g_H_. ***B***, Illustrative voltage traces of HTC cells from the stable region, particularly g_H_ = 0.36 mS/cm^2^ and unstable region (g_H_ = 0.22 mS/cm^2^).

**Figure 5.**
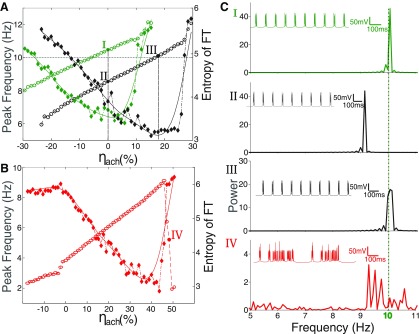
Rescue of the thalamic alpha with changing levels of basal [Ach] and HCN channel expression. ***A***, Increasing ambient ACh levels (*η_ach_*) increases the peak frequency of the rhythm (open circles, green for control HCN expression and open circles, black for I_H_ expression reduced to 80% of control). Peak frequencies remain lower than control when HCN expression is compromised to mimic known observations in AD (compare voltage traces shown in the insets of I and II in ***C***). The lowered frequency of alpha as a result of lower g_H_ can be rescued by increasing ACh levels. For example, 20% reduction in g_H_ (80% of control) needs ∼18% increase in ACh (see voltage trace in the inset of III in ***C***). There appears to be a threshold of ambient ACh levels beyond which entropy (filled diamonds; green for normal and black for reduced HCN expression) increases dramatically, suggesting a loss in periodicity. The overall entropy also remains high for decreased g_H_. ***B***, Severe reduction in HCN expression (45%, red) results in a lowered frequency of alpha and cannot be rescued by increasing ACh levels. With 45% g_H_ expression, HTC cells can achieve a maximum rhythm of 9 Hz before a complete breakdown of regular firing. This is seen as a sudden rise in entropy (filled red diamonds). ***C***, Power spectra corresponding to I, II, and III from ***A*** and IV from ***B***. Insets show the time series of the LFP corresponding to the Fourier transform.

In the temporal lobe of patients with sporadic AD, reduced expression of HCN channels has been reported (Saito et al., 2012). Further, suppressed HCN expression is also shown to accelerate amyloid-β aggregation in cell cultures (Neuro2a, a fast-growing mouse neuroblastoma cell line; Saito et al., 2012). Prompted by these observations, we investigate the precise effect of lowered HCN expression seen in AD on the occipital alpha rhythm. We varied the conductance of the I_H_ current (g_H_) to mimic the effect of changes in HCN expression in HTC cells (Fig. 4*A*). The control value for the conductance, set to 0.36 mS/cm^2^ (Vijayan and Kopell, 2012), generates 10-Hz rhythm. We quantified the periodicity of the rhythm using the power spectral entropy of the LFP (for details, see Materials and Methods). Higher values of entropy imply that the power in the signal is distributed over various frequencies. A periodic time series will exhibit low entropy, as the power would be confined to only a few narrow frequency regions (Fig. 5*CI*).

Reducing g_H_ over a limited range from 120% to 70% of the control value (0.36 mS/cm^2^), monotonically reduced the frequency of HTC firing (Vijayan and Kopell, 2012). Reduction in g_H_ has the effect of reducing the interburst interval of HTC cells (Fig. 2*C*). Peak frequency from power spectra is shown in Figure 4*A*, blue open circles. For g_H_ <0.27 mS/cm^2^ and >0.43 mS/cm^2^ (Fig. 4*A* at the boundary of the shaded region), periodicity breaks down and alpha rhythm is lost. This is seen as an increase in entropy (Fig. 4*A*, orange filled diamonds). Increased expression of I_H_ channels (higher value of g_H_) makes the membrane more excitable, because of which small fluctuations are more likely to cross the firing threshold, resulting in the noisy firing. Decreased expression of HCN, on the other hand, delays the onset of I_THT_ (Fig. 2). At values of g_H_ below 0.27 mS/cm^2^, the HTC cell membrane remains near the firing threshold over long periods of time. This makes the system sensitive to background noise which is now more likely to generate a supra-threshold perturbation leading to a spike. Our results show that the alpha rhythm is highly sensitive to HCN expression, as periodic activity of HTC cells is possible only over a limited regime of HCN expression.

### Limited rescue of the thalamic alpha with ACh

Increase in ambient ACh increases the excitability of HTC cells and initiates alpha. We hypothesize that, in early AD, reduced HCN expression in HTCs leads to lower excitability and delays the activation of the burst inducing calcium current. The chain of events that determine the timescale of the rhythm now take longer to complete. To understand the extent to which increased ACh levels can counter the effect of lowered HCN expression and recover the alpha rhythm. In Figure 5, different levels of HCN expression (green: normal; black: reduced to ∼80% of control) are shown and their effect on the alpha rhythm are described. We define *η_ach_* as a measure of the fractional change in cholinergic activity.

(5) $$\eta_{ach}=\frac{g_{Kleak\_norm}-g_{Kleak\_mod}}{g_{Kleak\_norm}}\times 100\;(\%).$$

Where *g_Kleak_*___ *_norm_* is the potassium leak conductance value that results in a 10-Hz alpha rhythm and *g_Kleak_*___*_mod_* is the modified potassium leak. Increase in ambient ACh levels (*η_ach_*) leads to a monotonic increase in the alpha frequency. Power spectra for healthy, pathologic and rescue cases of the alpha rhythm are shown in Figure 5*CI–III*. Increasing *η_ach_* by ∼20% from its normal value was able to rescue the alpha rhythm (Fig. 5*CIII*) in cells with reduced HCN expression (black, ∼80% of control). However, the increased excitability of HTC cells due to an increase in *η_ach_* also makes them more susceptible to noise. The loss of periodicity was quantified as an increase in power spectral entropy (filled diamonds). Here, we clearly see that rescue by ACh is possible only within a limited range of reduced HCN levels. For HCN levels as low as 45% of control (Fig. 5*B*) we observed that increases in ACh cannot bring the HTC rhythm up to 10 Hz and periodicity is restricted to frequencies below 9 Hz (Fig. 5*CIV*).

**Figure 6.**
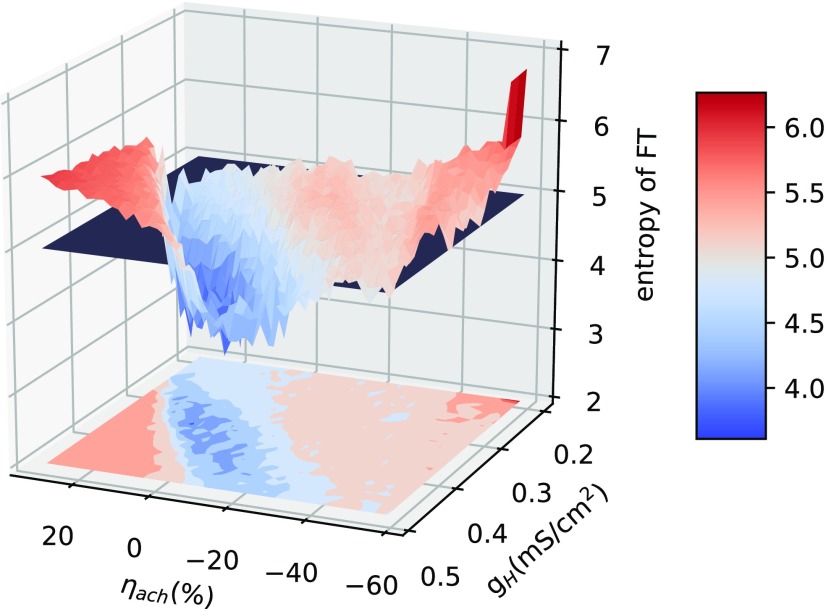
Parameter analysis for stable oscillations for a range of HCN expression and ACh levels. Higher entropy (red) implies aperiodicity in oscillations. The system transitions to aperiodicity at approximately entropy = 5.0 (demarcated by the black plane in the figure). There is a limited range of ACh and HCN expression over which stable oscillations are seen.

We explore a wide range of cholinergic tone (changing the potassium leak conductance) and HCN expression (g_H_) to find the range over which the network shows periodic oscillations. Periodic oscillations in the HTC cells are observed only over a limited regime of HCN expression (g_H_) and cholinergic tone (*η*; Fig. 6) marked by lower entropy (blue).

### Lowering g_H_ can lead to an enhanced GABA activity

**Figure 7.**
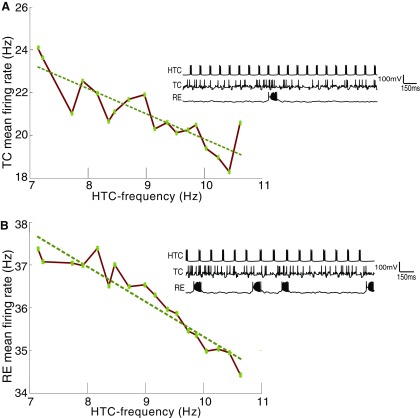
Increase in inhibitory activity follows a reduction in the firing rate of HTC cells. ***A***, There is an inverse relationship between HTC and TC firing. Lower drive from HTCs releases the inhibitory drive (via interneurons) on the TC cells causing an increase in their activity. Inset shows the time series of HTC, TC, and RE activity corresponding to a normal rhythm (10-Hz HTC frequency, g_H_ = 0.36 mS/cm^2^). ***B***, Inverse relationship between HTC firing and RE firing. Increased activity of the TC cells leads to enhanced RE activity. Inset shows the time series of HTC, TC, and RE activity corresponding to lowered HTC activity of 7.5 Hz due to reduced HCN expression (g_H_ = 0.28 mS/cm^2^).

Several studies have characterized modified occipital alpha in AD (Vitiello, 2015). Thus far we have shown how changes in ACh levels and HCN channel expression in the thalamus can modulate thalamic alpha (as the underlying basis for changes in occipital alpha). Next, we analyzed the effect of changing the alpha frequency, as seen in AD, on the dynamics of the thalamic network in our model. The decrease in the frequency of alpha was implemented as a decrease in I_H_ conductance (g_H_; Fig. 4*A*). This predictably leads to a lower frequency of HTC firing. Under these pathologic conditions of decreased HTC activity, the TC cells that were suppressed via GABAergic drive from the rhythm generating HTCs (Fig. 1) were released from inhibition. A corresponding increase in TC activity is seen in Figure 7*A*. The increase in TC activity causes the RE cells to fire at higher rates (Fig. 7*B*). The RE cells also receive excitation from HTC cells. The increase in RE firing rate happens despite the decreased drive from HTCs to the REs. This is because the HTC cells account for only 20% of the total excitatory drive to REs. The larger number of TCs (four times the number of HTCs) creates a positive feedback loop, causing an increase in RE activity. The enhanced activity of RE cells, in turn, enhances the inhibitory feedback to HTC cells reducing their frequency further (Fig. 7). The overall effect is thus an increase in GABAergic activity. While the increased presence of the neurotransmitter GABA has been reported in the astrocytes in the dentate gyrus (DG) of AD mice (Wu et al., 2014), we predict that lowered HCN expression can also tilt the balance between excitation and inhibition in the thalamic network. Our model suggests that lowered alpha rhythm frequency in the thalamus can cause a cascade of changes in thalamic network activity and may ultimately result in increased inhibition of HTC cells.

### Calcium current and its interaction with HCN expression in HTC cells

**Figure 8.**
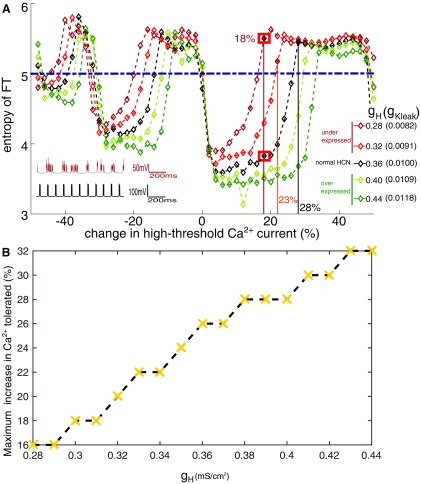
Lowered HCN makes the thalamic alpha more sensitive to small changes in the calcium conductance. ***A***, Colored lines show deviations from normal HCN expression levels. Systematically varying the calcium conductance in both directions leads to sudden increases in the entropy describing incoherent firing of the HTC cells. Lower HCN expression (g_H_ = 0.28 and 0.32 mS/cm^2^, maroon and red) have a lower tolerance for changes in calcium. This is seen as narrower windows of low entropy compared with the normal expression of HCN (g_H_ = 0.36 mS/cm^2^ black). Inset shows time series of the LFP when calcium conductance is enhanced by ∼18% for reduced HCN expression (maroon, g_H_ = 0.28 mS/cm^2^, irregular firing) and normal HCN expression (black, g_H_ = 0.36 mS/cm^2^, regular 10-Hz firing). Horizontal line (dashed dark blue) marks the threshold (5.0) for entropy beyond which we see irregular firing. ***B***, Tolerance to changes in calcium conductance decreases with decrease in HCN expression. For calcium conductance above the threshold value associated with each g_H,_ irregular firing is seen.

Elevated levels of cytosolic calcium linked to dysregulation in the calcium signaling are a common observation in AD neurons (Berridge, 2014). In order to simulate the pathology, we investigated the effect of changes in conductance of the high-threshold calcium current (g_THT_) that is crucial for the alpha rhythm, for a range of HCN expressions (g_H_; Fig. 8*A*; MacManus et al., 2000). The control system for each of the HCN expressions were initially set-up to generate 10-Hz frequency by appropriately changing the potassium leak conductance (see legend in Fig. 8*A*). Increased cytosolic calcium (pathologic state) corresponds to enhanced calcium entry via high-threshold calcium channels (corresponding to positive changes in g_THT_ in Fig. 8*A*). Interestingly, varying the conductance of the high-threshold calcium current does not change the frequency of the alpha rhythm significantly in our model. However, they can cause the HTC cells to lose coherence (Fig. 8*A*). As calcium conductance is changed, HTC cells go through regimes of periodic and irregular firing. These changes in periodicity can be seen in the sudden transitions in spectral entropy in Figure 8*A*. The burst of APs in HTC cells arises from the depolarizing current, of the high-threshold calcium channels (Fig. 2). After each AP, there is a competition between the amplitude of the hyperpolarizing potassium current and depolarizing calcium current, which determines whether the cell goes through another AP. The inactivation timescale of the calcium current dictates the number of APs in a burst. Increasing the conductance of the calcium current increases the duration over which APs can be generated. This is because it takes longer for the high threshold calcium current to decay to a value such that the potassium current can terminate the burst. This also implies that the amplitude of calcium current at the end of a burst for each “instantiation” of calcium conductance, will correspond to a different value of the potassium current. Thus, the number of APs in a burst increases with increasing conductance of the high-threshold calcium current. The regimes of chaotic activity illustrated in Figure 8*A* corresponds to the intermediate values of calcium conductance, as we go from *n* number of APs in a burst to *n* + 1 APs (range of n is 2–4). In these intermediate values of calcium conductance, the magnitudes of potassium and calcium currents are balanced at the end of the burst and background noise can randomly push the HTC cell toward another AP or hyperpolarize it to subthreshold voltages. This leads to periods of irregular firing for intermediate values of calcium conductance and non-monotonic dependence of periodicity on calcium current.

The range of calcium conductance over which regular alpha activity is maintained decreases with decreasing g_H_ (compare dark red with black in Fig. 8*A*). Healthy cells (g_H_ = 0.36 mS/cm^2^; Fig. 8*A*, black) appear robust and can tolerate as much as a 25% increase in the calcium conductance before losing periodicity. On the other hand, decreasing g_H_ values, to simulate the pathologic condition of lowered HCN expression shows lower tolerance for changes in calcium conductance. Figure 8*A* also describes the response to increased HCN expression (light green and dark green). The tolerance to increase in calcium is seen to systematically decrease with decrease in g_H_ (Fig. 8*B*). The heightened sensitivity to calcium is also seen as narrower troughs in the entropy (windows of regular firing) for the pathologic condition of lower expression of HCN (dark red and red traces in Fig. 8*A*). Lowering HCN expression (g_H_) corresponds to a systematic reduction in the tolerance to changes in calcium (summarized in Fig. 8*B*).

## Discussion

AD is a catastrophic disease that implicates multiple brain areas resulting in a range of symptoms. While precise molecular mechanisms that underlie the constellation of deficits and the causal links between them are not completely clear, several, apparently independent hypotheses have been proposed to delineate the root pathology. The most prominent of these relate to the toxic effects of accumulating amyloid-β plaques and Tau fibrils, a characteristic feature of AD (Binder et al., 2005). While amyloid-β and Tau-fibrils disrupt a wide array of signaling pathways in the brain, we do not yet have a complete understanding of the biochemistry that leads to their accumulation and proliferation. The calcium hypothesis of Alzheimer’s suggests that dysfunctional regulation of the calcium signaling profoundly vitiates neural functions like memory formation and consolidation by modifying synaptic plasticity and other signaling cascades. However, it is not known how minor changes in the calcium signaling cause drastic changes in behavior (Berridge, 2014). The cholinergic hypothesis proposes a reduced release of ACh as an early cause of the symptoms associated with AD (Drachman and Leavitt, 1974). In support of this, the most prevalent drugs administered to AD patients that provide temporary symptomatic relief are acetylcholinesterase inhibitors (Selkoe, 1997; Kumar et al., 2015) that increase the life span of ambient ACh. EEG studies report a reduction in power and frequency of the occipital alpha in a majority of AD patients as compared with control subjects (Vitiello, 1989; Hughes and Crunelli, 2005; Blinowska et al., 2017). Given the crucial role played by ACh signal in orchestrating occipital alpha (Lőrincz et al., 2009; Krishnan et al., 2016), these observations suggest compromised cholinergic signaling underlying its modification in AD.

### Analysis of causal relationships between HCN expression, alpha rhythm, and amyloid-β aggregation

Cognitive decline, including poor navigational ability, is observed before amyloid-β plaques are detected in AD (Allison et al., 2016). HCN expression in the entorhinal cortex regulates grid-cell formation and is essential for navigation (Giocomo et al., 2011). Interestingly it is also seen that drugs like Sildenafil, that enhance HCN activation, temporarily bring cognitive relief without affecting the amyloid-β load (Cuadrado-Tejedor et al., 2011). This suggests that compromised regulation in HCN expression can be one of the possible early changes triggering AD symptoms. HCN channels play a multitude of roles in the brain (Narayanan and Johnston, 2008; Honnuraiah and Narayanan, 2013) and aberrant expression of these channels have been associated with many pathologies (Herrmann et al., 2007; Blumenfeld, 2016). For a more comprehensive review of the role of HCN channels in neurodegenerative diseases see Chang et al. (2019). Observation of reduced HCN expression being correlated with over-expression of amyloid-β in AD neurons (Saito et al., 2012) is of particular interest to the present investigation.

**Figure 9.**
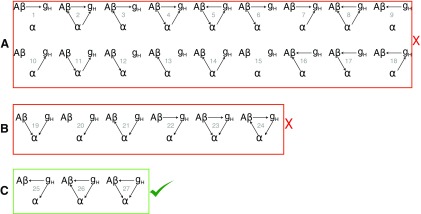
Potential causality between amyloid-β plaques (Aβ), HCN channels (g_H_), and the α rhythm (α). ***A***, HCN expression directly affects the thalamic α rhythm. Therefore, we can eliminate the possibilities where HCN expression does not affect the thalamic α rhythm. ***B***, The appearance of β-amyloid plaques and lowered expression of HCN channels are strongly correlated and therefore not independent of each other, in fact, lowered HCN expression has been shown to cause increased amyloid-β aggregation (Saito et al., 2012). We can eliminate all relationships where HCN expression does not affect amyloid-β. ***C***, Three possible causal relationships.

We explore the space of all possible causal relationships between amyloid-β (Aβ), HCN channel expression (g_H_) and modified thalamic alpha rhythm in AD. We have established that lowering the HCN channel expression, shown to be correlated with the occipital alpha rhythm, reduces the thalamic alpha rhythm frequency and coherence (Fig. 4; Hughes et al., 2011). The first box in Figure 9*A* lists all the possibilities, where I_H_ current channels (g_H_) do not disrupt the alpha rhythm. Given the insights from our model (a monotonic relationship between HCN expression and alpha peak frequency and coherence seen in Fig. 4), we can exclude these relationships. Postmortem studies of brains AD subjects have lower levels of HCN channels when compared with non-AD subjects (Saito et al., 2012). In studies where the HCN channels were knocked out (KO), Saito et al., report increased amyloid-β aggregation when compared with wild-type (WT) neurons (Saito et al., 2012). They also show that using an HCN channel blocker (ZD7288) in WT leads to similar levels of amyloid-β accumulation as the KO neurons. Statistically, these results suggest a relationship between reduced HCN expression and amyloid-β. Saito et al. (2012) also showed that HCN forms a complex with the amyloid-precursor protein (APP) *in vivo* and *in vitro*, suggesting a possible mechanism for HCN regulation of amyloid-β production. Using these observations, the relationships described in the second box which represents all other relationships in which HCN does not affect amyloid-β load can be eliminated (Fig. 9*B*). Our model combined with experimental observations reduces the list of relationships to merely three that are illustrated in the third box in Figure 9*C*. HCN channel expression affects both the alpha rhythm and amyloid-β directly. However, it is not yet clear if there is a directed link connecting amyloid-β and the alpha rhythm.

In light of the role of HCN expression in AD and the insights from the model, we propose that aberrant HCN expression may be a causal link to modified occipital alpha in AD. Downregulated HCN activity and expression may precede and possibly trigger excessive amyloid-β aggregation. AD is a multifaceted disease and ultimately invokes several cascading changes in the cells and extracellular biochemistry. We propose that HCN under-expression is one of the crucial early pathologies by which a regulatory breakdown can lead to excessive amyloid-β plaque build-up.

### Mechanisms for loss in coherence

**Figure 10.**
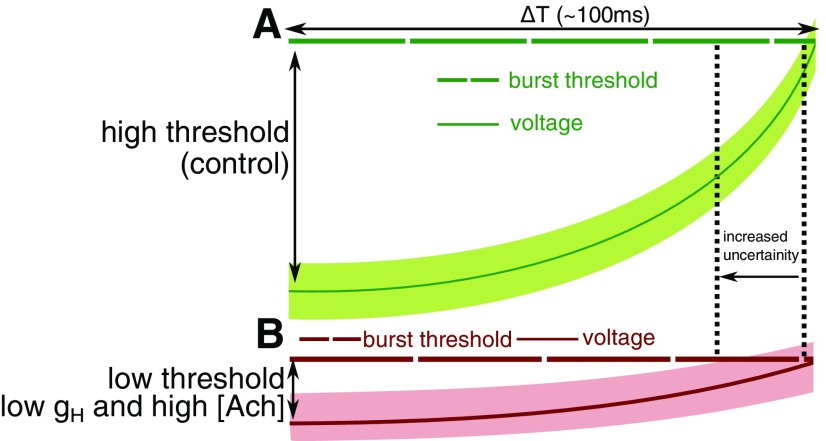
Illustration of the limited rescue of α rhythm. ***A***, Control: I_H_ current steadily depolarizes the membrane voltage (dark-green solid line) until it reaches the threshold for a burst (dark-green broken line). The light-green band around the voltage trace signifies the uncertainty in the membrane voltage due to noise. ***B***, Low g_H_ and high ACh: under these modified conditions the expected time taken for the voltage to reach the burst threshold is the same, but the noise, in this case, takes it closer to the burst threshold.

We characterize two distinct mechanisms that lead to a loss of coherence and lower power in alpha as observed in AD. (1) A loss of coherence with increased ACh is due to increased cell excitability. We demonstrate how excessive cholinergic modulation in the thalamic network leads to a sudden loss of coherence (Fig. 5*A*, green, *η_ach_* > 15*%*). Under these circumstances, a random background signal has a lower barrier to cross the threshold for initiating a burst, hence the exact time of initiating the burst underlying the alpha rhythm becomes unreliable. The same mechanism also underlies the loss in coherence with increasing g_H_ (Fig. 4, g_H_ > 0.43 mS/cm^2^). (2) Under control conditions (Fig. 10*A*), the HCN expression lies within a region where the cell does not spend too much time near the burst threshold, and at the same time has a substantial barrier to cross to reach the threshold. This allows for a robust 10-Hz burst to be precisely orchestrated that is predominantly unaffected by noise. Figure 10*B* illustrates how Reducing HCN expression (lower g_H_) reduces the rate at which the membrane depolarizes (Fig. 10*B*). The compensatory effect of increasing cholinergic tone can lower the threshold for initiating the burst to maintain the 10-Hz timescale of the rhythm (ΔT in the figure 10). However, this leads to an increased probability of noise initiating a burst imprecisely.

### Alpha Rhythm relation to overall firing rates and extracellular GABA

A recent study on transgenic mouse models of AD implicates enhanced GABAergic drive (Wu et al., 2014). In these studies, a temporary rescue of cognitive function was observed by reducing the inhibitory effect of GABA. Our model illustrates that higher GABA levels can be a direct downstream effect of lower frequency of the thalamic alpha rhythm due to reduced HTC firing frequency (Fig. 7). Reduction in HCN expression to mimic the AD pathology increases global firing rates. This is a consequence of the reduction in HTC firing frequency leading to a reduction in the inhibition on the TC cells. This, in turn, increases the activity in the GABA releasing RE cells (Fig. 7). Thus, a lower alpha will lead to increased activity of both TC and RE cells, this is consistent with the observation of normal alpha being associated with suppression of neuronal firing (Klimesch, 2012). In summary, our model predicts both an increase in overall activity and increased GABA levels in the thalamus under pathologic conditions of reduced alpha in AD.

## Conclusion

Using an alpha rhythm generating network model of the thalamus, we have systematically elucidated the causal links between different molecular changes associated with AD and their effect on the thalamic alpha. We hypothesize that HCN pathology causes thalamic alpha rhythm disruption, precedes amyloid-β plaque formation and may underlie early cognitive deficiency in the disease. Our results illustrate the limitations of therapeutic interventions that involve enhancing ACh. We predict the downstream effects of changes in thalamic alpha, leading to enhanced GABA activity. Mimicking increased calcium flux as seen in AD, results in global changes in network firing rate and a loss of coherence in the occipital alpha rhythm. When the HCN pathology is simulated, network activity becomes highly sensitive to small changes in calcium signaling and background noise. Often, disrupted brain rhythms are an early change associated with AD, our model can contribute to our understanding of early pathogenesis of the disease.

## Synthesis

Reviewing Editor: Tatyana Sharpee, The Salk Institute for Biological Studies Decisions are customarily a result of the Reviewing Editor and the peer reviewers coming together and discussing their recommendations until a consensus is reached. When revisions are invited, a fact-based synthesis statement explaining their decision and outlining what is needed to prepare a revision will be listed below. The following reviewer(s) agreed to reveal their identity: Giri Krishnan, Emmanuelle Tognoli.

The manuscript examines a computational model of thalamic network on how the change in cellular properties that likely to occur in Alzheimer’s disease impacts alpha oscillation. The authors use this to identify possible causal links between molecular changes and observable changes such as oscillatory activity and identify the limitations of pharmacological manipulation. Both these could have impact on clinical and pathophysiological studies on Alzheimer’s disease.

Reviewer 1:

In this manuscript, the authors used a detailed computational model of thalamic network to test the possible mechanisms for reduction in thalamic alpha rhythm in Alzhiemer’s disease. In particular, they show that reduced HCN expression results in reduction in thalamic alpha activity. More interestingly, the increase in cholinergic level had limited effect on mitigating this reduction. Based on the model, the authors provided possible hypothesis at the molecular level. Overall, the paper provides examination of mechanisms for reduction of thalamic alpha that can be linked specific molecular changes in AD.

There are several clarification about the work that would benefit the presentation of the results:

1. In the description of the chronology of events leading to thalamic alpha oscillation, the activity of neurons other than HCN is not shown. If this was a simulation of only HCN neuron, it need to be mentioned, and it would be helpful to show the evolution on the network. If other neurons were present in this simulation, it would be helpful to include them in the plot.

2. Given the number of HTC cells lower than the TC and RE cells, and TC cells project to cortex but not HTC cells, it may be better to compute LFP from TC cells.

3. One of the interesting result of this paper is that cholinergic increase only partially mitigated the reduction of alpha rhythm due to reduced HCN expression. Similar to the explanation of alpha oscillation generation, it would be helpful to provide mechanistic explanation of why acetylcholine had limited range in reduced HCN case. Part of the explanation is given in discussion, but would be helpful to explain this effect in greater detail. Also, it may be helpful to identify the region of stable alpha oscillations for different ach levels and plot it for different HCN expression.

4. It would be helpful to cite additional evidences for changes in HCN cells in AD. Other evidences of changes in thalamus may also indicate changes of HCN cells in thalamus, for eg. de Jong, Laura W., et al. “Strongly reduced volumes of putamen and thalamus in Alzheimer’s disease: an MRI study.” Brain 131.12 (2008): 3277-3285.

5. It is difficult to interpret the results of high-threshold Ca2+ current change only based on entropy. It would be helpful to include the change in power. Also it is not clear how the K+ leak was modified in the simulation, may be additional axis to show the values of K+ leak would be helpful. It is not clear why there is not a monotonic relationship between calcium conductance and alpha oscillation, is it due to the change in K+ leak ?, I think this section could help from better explaining the results.

6. It is very nice to identify the possible causal scheme based on the results of the computational model. How does the result about calcium in this causal scheme. Also, the fig can also indicate the different theories : cholinergic, calcium signalling and amyloid-beta.

Minor changes: In line 59, “muscarinic cholinergic agonization” could be better if modified as “muscarinic cholinergic activation”

Reviewer 2:

A. EXPOSITION OF MODEL’S EQUATIONS: I find the model exposition comprehensive but not friendly, especially to the less expert readership whose attention is drained trying to connect all the parts, principal and auxiliary alike. The front-end needs lot of details on the HTC cells (especially IL, IH, ITHT), and lesser on RE and TC cells. The current writing has every details of the model, and as a consequence of this density, we pass very quickly on key elements such as Acetylcholine in lns 143,184. I am wondering if a re-write exposing less details in “quantitative details of neurons and synapses”, and relegating other parameters to a supplementary material, would be a tractable solution.

B. THE MODEL’S FIXED PARAMETERS: I find myself wanting to know if all the fixed parameters were derived from model originators Vijayan & Kopell, or if some have been tuned by the authors of the manuscript and under which biophysical rationale, but this clarification is missing.

C. FIGURE FRIENDLINESS: This reviewer would find inclusion of legends in all figure inserts (not solely in the caption) much helpful to accelerate the grasp of their meaning: in the current form, much time is spent scanning from caption to figure and back trying to figure out who is who.

D. ENTROPY: the rationales, pros and cons of smoothing LFP traces is missing and seems important.

E. SITUATING THE COMPUTATIONAL MANIPULATIONS: In my opinion, it would be valuable to recontextualize the circuit model of figure 1 in the results figures 2-6 and/or to clearly state the locus of manipulation (HTC, TC, RE) in the caption’s title and in the section headers. In combination with (A.), this improvement will greatly aid the less specialized reader. Also, Figure 2 deciphers a chronology of events, and it would be valuable to add a conceptual diagram of the findings arisen.

F. A FEW MORE INSIGHT ON COLLECTIVE DYNAMICS? The study proposed a reduced model of HTC circuitry with only two neurons, an appropriately minimal model for the gap-junction that governs their behavior. This being said, two is more than one, and reading the findings raised several times some questions about the collective dynamics of those two cells, their coordination (especially, section “Effect of Varying HCN expression on thalamic alpha”, that entails hypothesis on both the loss of regularity within cell and the putative effect that their coherence has on mass action of the thalamocortical population as a whole), and its relation to the broaden alpha peak distribution in Figure 4. I suspect that such study might have happened to some degree (lns 351-352: “cause HTC cells to lose coherence”). Not imposing this extra body of work on the authors as a precondition for publication of their manuscript (I find the work deeper than most), I nonetheless thought to leave those burning questions with the authors in the eventuality of continuing research.

NOTES:

Title: could be sharpen a bit, this reviewer is not too fond of “AD-related pathologies”.

Abstract, the “downregulated frequency of alpha”: I find the word “downregulated” thick in multiple meanings and likely ambiguous in the context of alpha’s frequency decrease. Perhaps the authors would consider another choice of word?

Ln 127, eq. 1 reports ITRE but the following expose only provides a calcium current IT. Script error, or I do not understand the model?

Lns 127 and 169: IAPP seem underdefined since the sections “applied current” in lns 145-146 and 194-196 refer respectively to ulterior material, including equation 4 changing subscripts. Similarly, IGABAA and IAMPA of RE cells are elusive.

Ln 173: “The Sodium current [of HTC cells] follows the same dynamics as the potassium current in TC cells”. Is there a typographical error with potassium writing in place of Sodium?

Lns 184 and 196 forward mentions to posterior equations 6 and 4, which left this reviewer confused for a while, maybe adding the word “thereafter” would help?

Ln220 specifically says that HTC cells receive Poisson noise. It conflicts with earlier lns 113,195 speaking of a Gaussian White noise. The posterior explanation of why multiple Poisson processes can be modeled as a Gaussian (lns 222-225) is very clear and immediately corrects the confusion, but let me suggest a more careful choice of word to prevent questions arisen in the first place.

Figure 2: the top panel is not labeled, legends in all relevant inserts would be much helpful (comment C above), variables on the y-axis of panels C & F are difficult to relate to exposed model (see also comment A above for needed triage of important and less important aspect of the model).

Caption of Figure 2 could recall in the figure caption that study is taking place in HTC. The caption has an aggregate introduction to the left panel series A-C (between bursts) but misses on an equivalent introduction to the right panels D-F (within burst), degrading the legibility of the caption. Under (D), the color black for total current is not recalled. Similarly, under (F), the colors for IH and ITHT are not recalled, and force the reader to fish the meaning in the mirror graph C.

Ln273: combined with the looseness of the section header ln266, it was not immediately obvious to me where the H current was applied since in the model, H currents are found in TC and HTC cells (the answer is obvious to experts of HTC cells, however). Maybe a clarification would help, see also comment E above.

Ln 274: I am confused as to what is the conductance value. This line says 0.36, a color marker in figure 3 suggests same but ln187 says 0.4.

Ln 278: I might have issues with the choice of word coherent, usually reserved to speak of dyadic time series whose dynamics is correlated. In my understanding, here we talk about the temporal self-consistency of the cell’s bursting behavior. Perhaps, “periodic” would be more to the point?

Lns 286,312 and elsewhere. There are several instances of phrases stating “We see” where in fact the particular quantity is not plotted (therefore, we the reader, do not “see” along with the authors). It might be useful to spare the trouble for the reader to search for the inexistent plot, and just state that the results are “not shown”.

Figure 3: It might be useful to consider a multi-panel figure, showing ITHT. The orange plot in 3B shows a destabilized alpha bursting with excess HCN expression, but I understand that AD is related to under-expression, at the other end of plot A. Why not the latter?

Caption of Figure 4: There is a broken sentence under (B). The opening sentence speak of IH expression. Do you mean HCN expression?

Figure 5: The method used to derive the firing rate of TC and RE cells (y-axes), and with it, certain important nuances for the interpretation of the results, are missing.

Ln358: there is a fast and possibly slippery equivalence made between coherence and entropy.

Figure 6A: It might be helpful to enhance the legend of gH with specification of the parameter values that relate to under-expressed, normal and over-expressed HCN.

Caption of Figure 6 (A) identifies enhancement of calcium conductance by 20% but the line seems more like 18% and an annotation on figure says that much.

Ln 440 has a repeat of the word “the”.
